# Post-stroke Bruxism Improvement Following Botulinum Toxin Injection Into Salivary Glands: A Case Report

**DOI:** 10.7759/cureus.117962

**Published:** 2026-10-15

**Authors:** Amine Achraf Majit, Mohamed Harmouche, Mohammed Maarad, Ahmed Amine El Oumri

**Affiliations:** 1 Physical Medicine and Rehabilitation, Faculty of Medicine and Pharmacy, Mohammed First University, Oujda, MAR; 2 Rehabilitation Medicine, Provincial Hospital Center of Jerada, Jerada, MAR; 3 Intensive Care Unit, Faculty of Medicine and Pharmacy, Mohammed First University, Oujda, MAR; 4 Physical Medicine and Rehabilitation, Mohammed VI University of Health Sciences, Mohammed VI Foundation for Health Sciences, Rabat, MAR

**Keywords:** botulinum toxin a, masseter muscle, post-stroke bruxism, sialorrhea, ultrasound-guided injection

## Abstract

Sialorrhea and bruxism may occur after central nervous system injury and usually require separate management strategies. The effect of intraglandular botulinum toxin injection on bruxism is not well documented. A 37-year-old woman was admitted for rehabilitation after a right capsulolenticular hemorrhagic stroke. Four months later, she presented with left hemiplegia, dysarthria, dysphagia with aspiration, and severe sialorrhea, with a Drooling Severity and Frequency Scale (DSFS) score of 8/9. Awake bruxism characterized by tonic jaw muscle activity at rest was identified and graded 2 on the Standardised Tool for the Assessment of Bruxism (STAB), with tonic masseter activity on surface electromyography. Ultrasound-guided injections of onabotulinumtoxinA were performed in both parotid glands (30 units per side) and sublingual glands (20 units per side). At three weeks, sialorrhea improved with a DSFS reduction from 8/9 to 4/9. Bruxism decreased from STAB grade 2 to grade 1, with a reduction in masseter electromyographic activity. In this patient, intraglandular botulinum toxin injection for post-stroke sialorrhea was associated with improvement of bruxism. Further studies are required to clarify the relationship between salivary secretion and masticatory motor activity.

## Introduction

Following central nervous system injury, sialorrhea and bruxism may occur and may require different therapeutic approaches. Botulinum toxin type A can be used to reduce salivary production through injection into the salivary glands [1,2], whereas bruxism may be managed with behavioral interventions, occlusal devices, or botulinum toxin injections into the masticatory muscles [3]. Post-stroke sialorrhea is primarily related to impaired oral motor control, reduced swallowing frequency, and altered cortical control of salivary function [1]. Excessive saliva accumulation in the oral cavity increases the need for oro-motor clearance activity [2]. Bruxism, defined as repetitive jaw-muscle activity characterized by clenching or grinding of the teeth, or by bracing or thrusting of the mandible [4], may partially represent a compensatory motor response aimed at mobilizing pooled oral or oropharyngeal secretions [5,6,7]. Both conditions may share common neurological substrates. Cortical and subcortical lesions can alter the inhibitory control of brainstem motor circuits involved in salivary and masticatory functions [8,9], potentially contributing to their co-occurrence after stroke.

Despite this plausible neurophysiological relationship, the effect of modifying salivary flow on the severity of post-stroke bruxism has not been systematically investigated. The relationship between intraglandular botulinum toxin injection, modulation of salivary flow, and post-stroke bruxism remains poorly documented. This case report describes an improvement in post-stroke bruxism following intraglandular botulinum toxin type A injection for the treatment of sialorrhea. To our knowledge, the effect of intraglandular botulinum toxin injection for post-stroke sialorrhea on concomitant awake bruxism has not been previously reported. We hypothesized that reducing salivary secretion might be associated with a corresponding reduction in bruxism-related jaw muscle activity, given the shared neurophysiological substrates discussed above.

## Case presentation

A 37-year-old woman was admitted for inpatient rehabilitation four months after a right capsulolenticular hemorrhagic stroke of hypertensive origin. Neurological examination showed severe left hemiplegia graded on the Medical Research Council scale [10], spasticity of the left elbow flexors and knee extensors rated 2-3 on the Modified Ashworth Scale [11], dysarthria with tongue paresis, left central facial palsy, and dysphagia with aspiration confirmed by Fiberoptic Endoscopic Evaluation of Swallowing.

At admission, the patient was spatially oriented but partially disoriented to time, with psychomotor slowing and increased fatigability. She understood simple and complex verbal commands and participated appropriately in the rehabilitation program. Dysarthria remained intelligible, with preserved communication and no apparent word-finding difficulties. Examination also showed impaired buccofacial praxis, left spatial neglect, and left homonymous hemianopia. These findings were considered during rehabilitation and monitored clinically. No standardized cognitive, mood, or quality-of-life scales were administered during the three-week observation period.

From admission, she received multidisciplinary inpatient rehabilitation five days per week in 60-minute sessions: physiotherapy (postural control, trunk stabilization, transfer training, gait preparation, range-of-motion exercises, and spasticity management), occupational therapy (upper limb positioning, shoulder pain prevention, activities of daily living training, and caregiver education), and speech-language therapy (swallowing rehabilitation with compensatory strategies and dysarthria management), complemented by daily nursing-based positioning and swallowing precautions. Scopolamine transdermal patches (1.5 mg per 72 hours) had been used continuously for six weeks without adequate drooling control and were discontinued due to dry mouth and mild drowsiness; no further anticholinergic was prescribed given concern about cognitive side effects in the post-stroke context.

Sialorrhea was rated 8/9 (severity 4/5, frequency 4/4) on the Drooling Severity and Frequency Scale (DSFS) [12]. The Medical Research Council scale, Modified Ashworth Scale, and DSFS are freely available validated instruments used in accordance with their original publications [10-12]. Systematic history-taking from the patient and her family confirmed no prior jaw clenching, tooth grinding, jaw pain, temporomandibular joint symptoms, dental wear, or occlusal splint use before the stroke, supporting a neurological post-stroke origin for the jaw muscle overactivity. The identified phenotype was awake bruxism, characterized by sustained tonic masseter contraction during wakefulness without rhythmic grinding, assessed using the Standardised Tool for the Assessment of Bruxism (STAB). A STAB grade of 2 denotes probable bruxism requiring convergent clinical and electromyographic evidence; grade 1 denotes possible bruxism on clinical signs alone [13].

Surface electromyography was performed bilaterally over the masseter muscles using self-adhesive Ag/AgCl electrodes (10 mm diameter; 20 mm inter-electrode distance) along the longitudinal muscle axis, after light skin abrasion and cleansing with isopropyl alcohol. Signals were acquired at a bandwidth of 20-500 Hz, a sampling rate of 1000 Hz, and a common mode rejection ratio exceeding 80 dB over a 10-minute standardised resting recording with the patient seated and the jaw relaxed. Baseline surface electromyography findings were consistent with sustained masseter contraction during wakeful clenching and, combined with clinical examination, supported a STAB grade of 2.

Ultrasound-guided onabotulinumtoxinA injections were administered into both parotid glands (30 units per side) and both sublingual glands (20 units per side), totaling 100 units, with no injection into the masticatory muscles. At three weeks, sialorrhea had improved, with a DSFS reduction from 8/9 to 4/9, and awake bruxism had decreased, with a STAB grade reduction from 2 to 1, accompanied by a corresponding improvement in surface electromyography findings. No masseter weakness was reported; no chewing difficulty, reduced bite force, or jaw fatigue was identified, and no concomitant treatment was modified during the follow-up period. Baseline and three-week clinical findings are summarized in Table 1.

**Table 1:** Clinical findings before and after intraglandular botulinum toxin injection. Note: No specific licenses were required for the assessment tools used in this study, as they were utilized within the framework of routine clinical practice at our institution. DSFS: Drooling Severity and Frequency Scale; FEES: Fiberoptic Endoscopic Evaluation of Swallowing; MAS: Modified Ashworth Scale; MRC: Medical Research Council; sEMG: Surface electromyography; STAB: Standardised Tool for the Assessment of Bruxism

Parameter	Pre-intervention	Post-intervention (3 weeks)
Muscle strength: left lower limb (MRC)	Hip flexors, knee extensors, ankle dorsiflexors: 0–1/5	No clinically significant change
Muscle strength: left upper limb (MRC)	Shoulder abductors, elbow flexors, wrist extensors: 0/5	No clinically significant change
Spasticity (MAS)	Elbow flexors and knee extensors: 2–3	No clinically significant change
Dysphagia (FEES)	Laryngeal penetration and aspiration of thin liquids	No new aspiration events reported; FEES not repeated as the intervention did not target swallowing function
Tongue paresis	Present	Unchanged
Sialorrhea (DSFS)	8/9 (severity 4/5; frequency 4/4)	4/9 (severity 2/5; frequency 2/4)
Bruxism phenotype	Awake bruxism (sustained tonic clenching)	Same phenotype (tonic clenching), reduced intensity
Bruxism severity (STAB)	Grade 2: probable (clinical + sEMG confirmed)	Grade 1: possible (clinical signs only)
Masseter activity (sEMG)	Findings consistent with sustained clenching	Findings consistent with reduced clenching activity
Adverse events	None	None
Concomitant treatment	Unchanged	Unchanged

The clinical course and key therapeutic milestones from stroke onset through the three-week follow-up are summarized in Figure 1.

**Figure 1:**
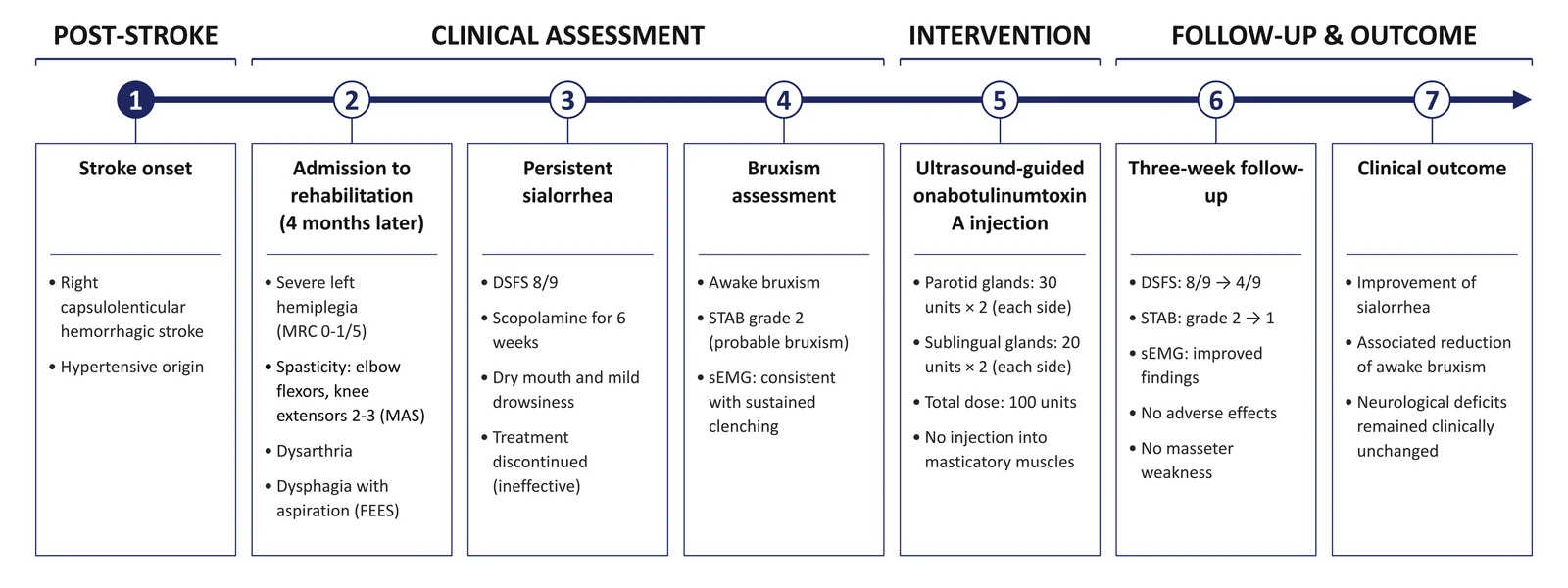
Clinical timeline of the patient's course from stroke onset to three-week follow-up after ultrasound-guided intraglandular onabotulinumtoxinA injection. The timeline summarizes the major clinical events, including stroke onset, admission to rehabilitation, persistent sialorrhea despite six weeks of transdermal scopolamine therapy, identification of awake bruxism by clinical examination and sEMG, ultrasound-guided onabotulinumtoxinA injection into the parotid and sublingual glands, and three-week follow-up demonstrating improvement in both sialorrhea and awake bruxism without treatment-related adverse effects. DSFS: Drooling Severity and Frequency Scale; FEES: Fiberoptic Endoscopic Evaluation of Swallowing; MAS: Modified Ashworth Scale; MRC: Medical Research Council; sEMG: Surface electromyography; STAB: Standardised Tool for the Assessment of Bruxism

Written informed consent was obtained. This report was prepared in line with the CARE 2013 criteria [14].

## Discussion

Botulinum toxin type A injection into the salivary glands reduces salivary flow and is used to manage neurogenic hypersalivation [1]. Management of bruxism includes behavioral interventions, occlusal devices, and injections into masticatory muscles when necessary [3].

Reports describing improvement of bruxism after intraglandular injection are limited. In patients with Rett syndrome, bruxism decreased after salivary gland injection without muscle involvement [5]. By contrast, high-dose intraglandular injections (up to 250 units) did not alter chewing or swallowing, suggesting that the effect is limited to the glands [15].

The bruxism observed in this patient was consistent with the awake phenotype as defined by current international consensus [4,13], characterized by sustained tonic masseter contraction during wakefulness rather than sleep-related rhythmic grinding. Crucially, no prior history of bruxism either self-reported or inferred from dental findings was identified, supporting a neurological, post-stroke etiology. This distinction is clinically relevant because pre-existing bruxism would confound the interpretation of any treatment effect.

In post-stroke patients, disruption of cortical and subcortical inhibitory control over brainstem circuits governing salivary secretion [8] and masticatory pattern generation [16] may disinhibit both systems, predisposing to excessive salivary pooling and involuntary jaw clenching. The co-occurrence of severe sialorrhea in this context raises the possibility that excess pooled oral secretions further activate trigeminal afferent pathways, amplifying this motor dysregulation, and suggesting that modifying oropharyngeal sensory input may in turn influence involuntary masticatory activity [9,16].

Bruxism may act as an adaptive motor response to mobilize stagnant saliva [5,6]. Polysomnographic studies suggest that changes in salivary flow can modulate rhythmic masticatory activity [6]. Salivary cortisol levels also correlate with bruxism, indicating a neurophysiological link between salivation, stress, and masticatory activity [17].

The hypothesis of an adaptive role for bruxism as a salivary clearance mechanism is supported by the work of Miyawaki et al. [7], which showed a correlation between rhythmic activity of the masticatory muscles and the swallowing reflex. The adaptive bruxism-salivation link remains speculative and requires prospective confirmation.

Several alternative explanations for the observed improvement in bruxism must be considered. First, spontaneous neurological recovery is a well-recognized phenomenon in the months following stroke and cannot be excluded as a contributing factor, particularly given that the intervention occurred four months post-onset, a period during which partial functional restitution may still occur. Second, the temporal association between sialorrhea reduction and bruxism improvement, while suggestive, does not establish causality; indirect effects such as improved overall comfort, reduced aspiration events, or changes in the patient's level of arousal and anxiety related to drooling may have independently reduced jaw clenching. Third, the STAB tool grades bruxism based on a combination of clinical and electromyographic criteria [13]. Although the reduction from grade 2 to grade 1 was clinically relevant, it occurred at the boundary between probable and possible bruxism and should therefore be interpreted cautiously. Surface electromyography is also subject to inter-session variability related to electrode placement, skin impedance, muscle activation, and physiological fluctuations [18]; the observed reduction in masseter activity should therefore be considered supportive of clinical improvement rather than definitive evidence of a change in bruxism severity.

An alternative explanation for the decrease in bruxism could be unintended diffusion of botulinum toxin into the masseter muscle due to its anatomical proximity to the parotid gland. However, ultrasound guidance allows visualization of glandular and muscular planes, which reduces the risk of intramuscular injection [19]. In addition, no signs of masseter weakness were observed. Masseter weakness secondary to botulinum toxin diffusion would be expected to manifest as difficulty initiating chewing, reduced bite force, or jaw fatigue, none of which were reported. While ultrasound guidance substantially reduces the risk of intramuscular spread, it does not eliminate it entirely; subclinical toxin diffusion affecting afferent spindle signaling within the masseter without producing clinically detectable weakness remains a theoretically plausible mechanism that cannot be fully excluded on the basis of clinical assessment alone [20].

This case report has several limitations. As a single-case observation, the findings are descriptive and hypothesis-generating and cannot be generalized. The three-week follow-up does not allow assessment of the long-term durability of the observed improvement or the potential need for repeated injections. Formal standardized assessments of mood, anxiety, and quality of life were not performed, and their potential contribution to the observed clinical improvement cannot be excluded. The sEMG findings should also be interpreted in light of the inter-session variability discussed above.

## Conclusions

In this patient, treatment of post-stroke sialorrhea with ultrasound-guided intraglandular onabotulinumtoxinA was followed by an improvement in both sialorrhea and awake bruxism, with corresponding changes in clinical and electromyographic findings. The concurrent improvement of these two conditions suggests that salivary function may be linked to masticatory motor activity after stroke, although this association does not establish a causal relationship. This observation highlights a possible role for salivary modulation in the management of post-stroke awake bruxism. Larger controlled studies are required to determine whether modification of salivary secretion influences involuntary masticatory activity after stroke.

## References

[1] Jost WH, Bäumer T, Laskawi R (2019). Therapy of sialorrhea with botulinum neurotoxin. Neurol Ther.

[2] Lakraj AA, Moghimi N, Jabbari B (2013). Sialorrhea: anatomy, pathophysiology and treatment with emphasis on the role of botulinum toxins. Toxins (Basel).

[3] Jabłońska O, Galanty-Ochyra A, Nosal A (2025). From risk factors to treatment: a holistic approach to managing bruxism. J Educ Health Sport.

[4] Lobbezoo F, Ahlberg J, Glaros AG (2013). Bruxism defined and graded: an international consensus. J Oral Rehabil.

[5] Bernardo P, Raiano E, Cappuccio G (2019). The Treatment of Hypersalivation in Rett Syndrome with Botulinum Toxin: Efficacy and Clinical Implications. Neurol Ther.

[6] Shinkai M, Suzuki Y, Ozawa A, Shibagaki A, Okura K, Matsuka Y (2026). Associations between sleep bruxism, oral wetness, and salivary flow: A quantitative analysis. J Prosthodont Res.

[7] Miyawaki S, Lavigne GJ, Pierre M, Guitard F, Montplaisir JY, Kato T (2003). Association between sleep bruxism, swallowing-related laryngeal movement, and sleep positions. Sleep.

[8] Proctor GB (2016). The physiology of salivary secretion. Periodontol 2000.

[9] Kim HK, Chung KM, Xing J, Kim HY, Youn DH (2024). The trigeminal sensory system and orofacial pain. Int J Mol Sci.

[10] Medical Research Council (1976). Aids to the examination of the peripheral nervous system.

[11] Bohannon RW, Smith MB (1987). Interrater reliability of a modified Ashworth scale of muscle spasticity. Phys Ther.

[12] Thomas-Stonell N, Greenberg J (1988). Three treatment approaches and clinical factors in the reduction of drooling. Dysphagia.

[13] Manfredini D, Ahlberg J, Aarab G (2024). Standardised tool for the assessment of bruxism. J Oral Rehabil.

[14] Gagnier JJ, Kienle G, Altman DG, Moher D, Sox H, Riley D (2013). The CARE guidelines: consensus-based clinical case reporting guideline development. J Med Case Rep.

[15] Mueller J, Langbein T, Mishra A, Baum RP (2022). Safety of high-dose botulinum toxin injections for parotid and submandibular gland radioprotection. Toxins (Basel).

[16] Morquette P, Lavoie R, Fhima MD, Lamoureux X, Verdier D, Kolta A (2012). Generation of the masticatory central pattern and its modulation by sensory feedback. Prog Neurobiol.

[17] Karakoulaki S, Tortopidis D, Andreadis D, Koidis P (2015). Relationship between sleep bruxism and stress determined by saliva biomarkers. Int J Prosthodont.

[18] Hermens HJ, Freriks B, Disselhorst-Klug C, Rau G (2000). Development of recommendations for SEMG sensors and sensor placement procedures. J Electromyogr Kinesiol.

[19] So JI, Song DH, Park JH, Choi E, Yoon JY, Yoo Y, Chung ME (2017). Accuracy of ultrasound-guided and non-ultrasound-guided botulinum toxin injection into cadaver salivary glands. Ann Rehabil Med.

[20] Hok P, Veverka T, Hluštík P, Nevrlý M, Kaňovský P (2021). The central effects of botulinum toxin in dystonia and spasticity. Toxins (Basel).

